# SVR12 Higher than 97% in GT3 Cirrhotic Patients with Evidence of Portal Hypertension Treated with SOF/VEL without Ribavirin: A Nation-Wide Cohort Study

**DOI:** 10.3390/cells8040313

**Published:** 2019-04-04

**Authors:** Alessandra Mangia, Giovanni Cenderello, Massimiliano Copetti, Gabriella Verucchi, Valeria Piazzolla, Celeste Lorusso, Rosanna Santoro, Maria Maddalena Squillante, Alessandra Orlandini, Rosalba Minisini, Alessia Ciancio

**Affiliations:** 1 Liver Unit, Fondazione IRCCS “Casa Sollievo della Sofferenza”, 71013 San Giovanni Rotondo, Italy; valeria.piazzo@libero.it (V.P.); lorusso.celeste590@gmail.com (C.L.); r.santoro@operapadrepio.it (R.S.); squillante.ma@tiscali.it (M.M.S.); 2 Infectious Disease Unit, Ospedale “Galliera”, 16128 Genova, Italy; giovanni.cenderello@galliera.it; 3 Biostatistics Unit, Fondazione IRCCS “Casa Sollievo della Sofferenza”, 71013 San Giovanni Rotondo, Italy; m.copetti@operapadrepio.it; 4 Infectious Disease Unit, Università di Bologna, 40126 Bologna, Italy; gabriella.verrucchi@libero.it; 5 Infectious Disease Unit, University of Parma, 43125 Parma, Italy; aorlandini@ao.pr.it; 6 Internal Medicine, Università del Piemonte Orientale, 28100 Novara, Italy; rosalba.minisini@med.unipo.it; 7 Gastroenterology Unit, Università di Torino, 10124 Torino, Italy; alessiaciancio@libero.it

**Keywords:** HCV, SOF/VEL, genotype 3, cirrhosis, portal hypertension

## Abstract

In clinical trials, a sofosbuvir/velpatasvir (SOF/VEL) pangenotypic single-tablet regimen was associated with high sustained virological response (SVR) rates at 12 weeks (SVR12) after the end of treatment, regardless of genotype and fibrosis stage. No real-life data on genotype 3 (GT3) cirrhotic patients with portal hypertension are available. The aim of this study was to assess the effectiveness of SOF/VEL in GT3 cirrhotics with portal hypertension. Patients with GT3 and advanced cirrhosis were treated for 12 weeks with SOF/VEL without ribavirin at five different centers in Italy from June 2017 to August 2018 and their SVR12 was assessed. Of the 227 GT3 cirrhotics evaluated, 205 met the inclusion criteria and 111 had transient elastography results ≥20 KPa. SVR12 was 97.6% (95% CI 94.4–98.9), rates were 99.1% (95% CI 95.7–99.8) in patients with ≥20 KPa and 95.8% (95% CI 89.5–98.3) in those with <20 KPa (*p* = 0.18). Analyzed by presence of esophageal varices, the SVR12 rates were 98.4% (95% CI 91.4–99.7) and 97.1% (95% CI 92.9–98.9) in patients without and with varices, respectively (*p* = 1.0). In real life, SOF/VEL GT3 cirrhotic patients with evidence of portal hypertension can achieve SVR12 levels comparable to those of patients without portal hypertension. These SVR12 rates are similar to what is reported in compensated cirrhosis treated within clinical trials.

## 1. Introduction

In the era of directly acting antivirals (DAA) treatment, despite advances in global sustained virological response at 12 weeks (SVR12) rates, patients with genotype 3 (GT3) infection and cirrhosis have emerged as a difficult-to-cure population [1].

The single-tablet regimen (STR) of sofosbuvir (SOF) (nonstructural protein 5B (NS5B) inhibitor) and velpatasvir (VEL) (nonstructural protein 5A (NS5A) inhibitor) for 12 weeks demonstrated high efficacy across all genotypes in clinical trials [2,3]. These results were confirmed in real life [4,5]. Improvements in patient outcomes in clinical studies were also shown in studies dedicated to GT3 alone, including ASTRAL-3 and POLARIS-3 with SVR12 rates of 95% and 96%, respectively [3,6,7]. Real-world data in a well-characterized population of GT3 patients with cirrhosis are still limited.

A large real-world cohort of GT3 compensated cirrhotic patients from different countries around the world was recently analyzed [8]. This post hoc analysis confirmed the high efficacy of SOF/VEL without ribavirin in patients with cirrhosis and different baseline characteristics including patients with transient elastography (TE) results ≥20 KPa. This TE threshold was proposed together with platelet (PLT) count <150,000 µL at the Baveno VI international workshop to avoid unnecessary endoscopic screening and the surveillance of variceal bleeding in cirrhotic patients with a low likelihood of portal hypertension [9]. However, a more detailed characterization of portal hypertension, including esophageal or gastric varices and/or hypertensive gastropathy, was not available in that or in other studies focusing on treatment. In addition, the outcome of patients with more advanced liver disease receiving all-oral antiviral therapy, and in particular those with clinically significant portal hypertension, is under investigation [10,11]. In a multicenter prospective study of patients with hepatitis C virus-associated cirrhosis, an SVR to all-oral therapy was shown to be associated with significant hepatic venous pressure gradient (HVPG) reduction, compared with before treatment [11].

The use of ribavirin with the SOF/VEL STR combination has been largely debated. In patients with decompensated cirrhosis, based on the ASTRAL-4 results [12], all international guidelines recommend the addition of ribavirin (RBV) to SOF/VEL regardless of hepatitis C virus (HCV) genotype.

Discordant advice is provided by European Association of Sinological Librarians (EASL) and American Association for the Study of Liver Diseases (AASLD) in patients with compensated cirrhosis. Indeed, European guidelines recommend the addition of ribavirin in GT3 cirrhotic patients with prior treatment experience [13,14]. This recommendation is mostly driven by the risk of Y93H substitution (resistance associated substitutions (RASs)) in the HCV NS5A region associated with resistance to velpatasvir [13]. However, a recent phase 3 trial of 204 patients with GT3 and compensated cirrhosis did not show a significant difference in SVR12 rates for GT3 cirrhotic patients treated with or without RBV [15]. 

Patients with compensated cirrhosis and grade 1 and 2 never bleeding esophageal varices represent a subgroup of patients with a better prognosis compared to patients in Child–Pugh–Turcotte (CTP) class B despite having an expected higher mortality than uncomplicated CTP class A. As shown by a recent analysis, identifying five prognostic stages of cirrhosis, patients with compensated cirrhosis and varices have a 5-year mortality rate of 10% as compared to the 1.5% observed in patients with compensated cirrhosis and an absence of varices [16].

Analyses of SVR12 after DAA treatment in patients with cirrhosis and varices have not yet been performed and might provide further insights helping with the individualization of the treatment of patients with cirrhosis.

Since a chasm between efficacy (in clinical trials) and effectiveness (in clinical practice) persists, in particular in patients with unfavorable characteristics, we explored in a multicenter nationwide study the impact of SOF/VEL treatment in cirrhotic patients with or without evidence of portal hypertension treated without the addition of ribavirin.

## 2. Methods

### 2.1. Patients

Patients with GT3 and cirrhosis enrolled at five different tertiary centers in Italy and treated from June 20, 2017 to August 20, 2018 with SOF/VEL 400/100 mg STR for 12 weeks without the addition of ribavirin. Eligible patients were males and females, 18 years of age or older with confirmed chronic GT3 infection and compensated cirrhosis. Patients with CTP class C and prior-treatment experience (including NS5B inhibitors, NS5A and NS3/4 protease inhibitors) were excluded. Patients with previous decompensation and patients with malignancies were included. Patients being treated in those centers with the addition of ribavirin as well as patients that were treated for longer than 24 weeks were excluded. Patients with human immunodeficiency virus (HIV) or HBs Ag were considered eligible if hepatitis B virus DNA (HBV DNA) was not reactive.

### 2.2. Assessments 

Serum HCV RNA levels were measured using commercial HCV RNA quantitative assays. HCV RNA was defined unquantified when below the lower limit of quantification (LLOQ) and unquantified but detectable when 12 IU/mL or 15 IU depending on the quantitative assays used. Viremia load was defined as high when it was higher than 1,000,000 IU/mL. The aspartate/platelets ratio index, AST to platelet ratio index (APRI) score and fibrosis-4 (FIB-4) were calculated for every patient. Cut-offs of >2.0 and >3.25 were respectively used to define cirrhosis [17]. Diagnosis of the fibrosis stage was based on liver biopsy or TE (FibroScan, Echosens, Paris, France) results using the standard threshold of 12.5 KPa to define cirrhosis and 10.1 to define advanced fibrosis or on non-invasive biochemical indices when TE was not applicable. Patients with ascites were evaluated only by APRI and FIB-4 scores; all of the remaining patients were evaluated by TE plus APRI and FIB-4.

Portal hypertension absence or presence was estimated by transient elastography results <20 KPa or ≥20 KPa and platelet count <150,000 µL. [9]. Upper abdominal ultrasound was performed at baseline, searching for a spleen diameter >15 cm or portal vein (PV) diameter >15 mm in all patients and upper endoscopy was performed only in patients with TE results higher than 20 KPa. A combination of non-invasive markers including ultrasound (US) signs of portal hypertension and clinical data including ascites and previous history of decompensation were considered to confirm non-invasive diagnosis of portal hypertension in the group of patients with high TE results.

Adverse events and laboratory tests were conducted during this study, and at week 12 post-treatment for a safety evaluation. Monitoring and grading of adverse events (AEs) and serious AEs (SAEs) were performed. Evidence of esophageal or gastric varices and evidence of hypertensive gastropathy confirmed portal hypertension. Ultrasonographic evidence of portal hypertension was based on the combination of spleen longitudinal diameter ≥15 cm and portal vein diameter ≥14 mm.

### 2.3. Endpoints 

The primary efficacy endpoint was SVR12 (HCV RNA < LLOQ 12 weeks after the end of therapy). Patients with missing SVR12 data were considered a treatment failure. The primary safety endpoint was discontinuation due to AEs.

### 2.4. Statistical Methods

Patients’ demographical and clinical characteristics were reported as mean and standard deviation or as median and range and as frequency and percentages for continuous and categorical variables, respectively. Group comparisons were performed using Mann–Whitney U-test or Fisher exact test for continuous and categorical variables, respectively.

The SVR rates were assessed overall and in subgroups as defined by demographical and clinical variables. SVR rates by treatment regimen and treatment experience were also analyzed. Confidence intervals (CIs) of 95% for SVR rates were assessed using the binomial distribution. A *p*-value <0.05 was considered as statistically significant. Statistical analyses were performed using SPSS (IBM, Armonk, NY, USA) v. 16.0.

## 3. Results

### 3.1. Patients

**Figure 1:**
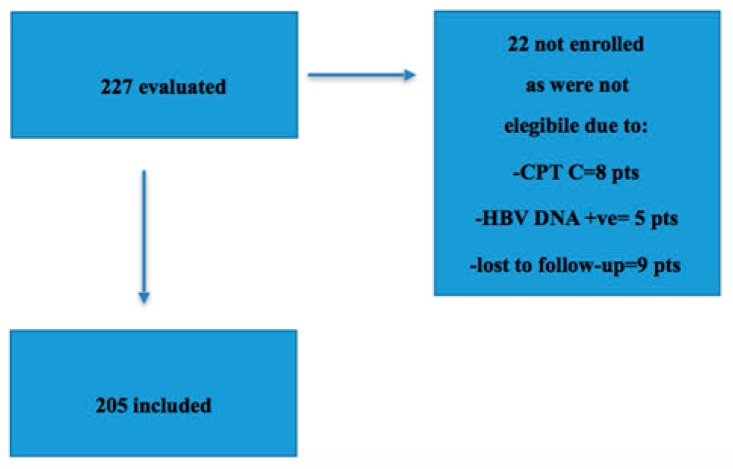
Study flow-chart. Of 227 patients with genotype 3 (GT3) and cirrhosis evaluated for treatment at five tertiary or academic centers in Italy, 205 patients with cirrhosis started treatment with sofosbuvir/velpatasvir (SOF/VEL) without ribavirin. Eight patients had concomitant active hepatitis B virus (HBV) infection and five had a decompensated cirrhosis. Nine patients were lost to follow-up before the start of treatment.

Of the 227 patients evaluated, only 205 with HCV GT3 and compensated cirrhosis with or without evidence of portal hypertension were included. They were treated with SOF/VEL, STR for 12 weeks without RBV. Of the 22 who were not enrolled, eight had CTP class C, five had active HBV DNA replication that required a delay in treatment start and nine were lost to follow-up (Figure 1).

Patients’ baseline characteristics are summarized by overall and by TE results (Table 1). The mean age was 52.9 ± 8.7 years, the vast majority were male, and all were Caucasian. Almost all patients had GT3 of subtype a, and TE ranged from 12.5 to 75.0 KPa. About 20% of patients had evidence of diabetes or past or current alcohol abuse, 15% had a history of intravenous drug use, and 10% were HIV-positive. Less than 20% had a previous treatment experience based on the Peg-Interferon and RBV regimen.

Overall, 111 patients had TE results that were >20 KPa; clinical characteristics are reported in Table 1. This subgroup of patients, considered to be at high risk of portal hypertension (8), had esophageal or gastric varices or hypertensive gastropathy in almost 60% of cases. Of 63 patients with esophageal or gastric varices or hypertensive gastropathy, 41 had esophageal varices F1, 13 F2 and one F3. Severe hypertensive gastropathy was observed in five cases and gastric varices in three.

A PLT count of <150,000 µL was observed in almost 65.3% of cases. Among patients with high TE results, 10 had hepatocellular carcinoma (HCC). Two patients had non-Hodgkin’s lymphomas and both of them had TE results <20.0 KPa. Overall, 35 patients had a spleen longitudinal diameter of 15 cm or higher and a PV diameter >15 mm. Of them, 29 had TE results >20 KPa, while the remaining were shown to have a lower value. Mild ascites was detected in eight patients.

### 3.2. Efficacy Analysis Overall and by Treatment History

After 12 weeks of treatment, SVR12 was 97.6% (95% CI 94.4–98.9) overall and no virological failures were observed. Overall, five patients experienced a virological relapse (Table 2). No loss to follow-up or treatment discontinuations was registered. In order to increase SVR12, the use of ribavirin was advised in cirrhotic patients with GT3 and prior treatment failure, patients were analyzed by treatment history. Despite the absence of RBV, SVR12 was 98.0% (95% CI 94.2–99.3) in naïve and 96.4% (95% CI 86.5–98.9) in prior Pegylated Interferon (PegIFN) failures (*p* = 0.61).

### 3.3. Efficacy Analysis by Severity of Liver Disease and Evidence of Portal Hypertension

**Figure 2:**
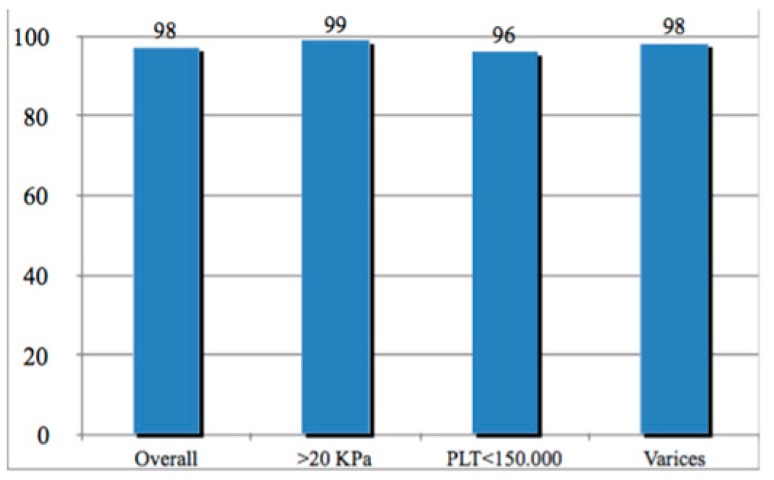
Sustained virological response at 12 weeks (SVR12) overall and by portal hypertension. SVR12 was extremely high regardless of baseline characteristics of patients including portal hypertension-associated features as TE >20 KPa, platelet (PLT) count <100,000 mm^3^ and evidence of varices or hypertensive gastropathy.

Upon analysis by TE results ≥20 KPa, SVR12 was 99.1% (95% CI 95.7–99.8) compared to 95.7% (95% CI 89.5–98.3) registered in patients with lower KPa results (*p* = 0.18). Among those with TE results ≥20 KPa (64.5%), 62 of 96 patients who underwent upper endoscopy had endoscopic evidence of portal hypertension and only one patient had a relapse (Figure 2). By evidence of varices or hypertensive gastropathy, SVR12 was 98.2% (95% CI 91.4–99.7) in 63 patients with varices and 97.1% (95% CI 92.9–98.9) in 142 patients without varices (*p* = 1.0).

Overall, 64 patients had ultrasonographic evidence of portal hypertension, SVR12 in this subgroup was 98.4% (95% CI 91.7–99.7). This response rate was not different from the 97.0% (95% CI 92.6–98.8) SVR12 attained in patients without ultrasonographic signs of portal hypertension (*p* = 1.0).

Having identified the threshold of the PLT count of 150,000 µL as highly suggestive of portal hypertension, patients were analyzed accordingly. SVR12 was 99.2% (95% CI 95.7–99.8) for 133 patients with lower PLT counts and 94.4% (95% CI 86.5–97.8) for 72 patients with higher PLT count results (*p* = 0.53).

Albumin levels lower than 3.5 g/dL were registered in 30 patients. All of them achieved SVR12, in contrast with 170 of 175 with higher albumin levels (SVR12 97%) (95% CI 93.4–98.7) (*p* = 1.0). Of the 22 patients who had both low albumin levels and a low PLT count, 100% achieved SVR12.

Finally, patients were also analyzed by CTP score A5 versus higher scores. In 162 patients with CTP A5, SVR12 was 98.1% (95% CI 94.7–99.3) as compared to 95.3% (95% CI 84.5–98.7) in 43 patients with higher CTP scores (*p* = 0.28).

In patients with ascites, SVR12 was 88.9% (95% CI 56.5–98.0) as compared to 95.3% (95% CI 84.5–98.7) achieved in patients without ascites (*p* = 0.23). In 10 patients with HCC, SVR12 was 100% as compared to 96.2% (95% CI 93.4–98.5) achieved in patients without HCC (*p* = 1.0).

### 3.4. Efficacy by Co-Morbidities

No difference is SVR rate was observed in patients with or without alcohol abuse history, past history of intravenous drug use or HIV co-infection.

All 36 patients with diabetes achieved SVR12.

### 3.5. Safety

Overall SOF/VEL was well tolerated. No patients prematurely discontinued treatment due to AEs. The most frequently reported AEs were depressive syndrome, dizziness and anxiety. AEs were mild and the most common adverse event was asthenia (28% of patients). Headaches were registered in 15%, insomnia in 10% and diarrhea and abdominal pain in 3% of patients during treatment.

SAEs were rare; three patients had urinary tract infections, two had tracheobronchitis and one had bronchopneumonitis. No SAEs events were considered to be related to the antiviral treatment. Three patients died after follow-up completion, one of lung cancer, one of worsening liver disease and ascites complication and another of HCC.

Laboratory abnormalities were infrequent. Few patients experienced anemia (2%) and 1.4% experienced an increase in bilirubin levels.

## 4. Discussion

In this multicenter prospective real-world cohort, GT3-infected patients with cirrhosis, half of whom exhibited the presence of portal hypertension by non-invasive evaluation, achieved an SVR12 rate of 97.6%. SVR12 rates of 99.1% were observed in the subgroup of patients considered at high risk of portal hypertension.

Randomized controlled trials demonstrated that SVR12 rates were associated with SOF/VEL range from 91% in the ASTRAL-3 study to 93% in a combined analysis of the 212 GT3 patients with compensated cirrhosis included in the ASTRAL program, and to 96% observed in the POLARIS-3 trial [3,6,18]. No details on portal hypertension were available in the registration studies. The response rate of our study is consistent with those of ASTRAL-3 and POLARIS-3 trials [3,6], although compared to the subgroup of patients with compensated cirrhosis that were included in the mentioned trials, our patients were older and had a higher prevalence of co-morbidities. Moreover, our patients had compensated cirrhosis with TE results ≥20 KPa and PLT counts <150,000 µL/mm^3^, suggestive of portal hypertension [9,19], or endoscopic and abdominal ultrasound results consistent with portal hypertension. These results demonstrated high effectiveness in real-world situations, despite ribavirin not having been included in the treatment regimen.

The effectiveness observed in our cohort is comparable to that reported in a multinational cohort on over 400 GT3 patients with compensated cirrhosis from around the globe. That study compared SOF/VEL to SOF/VEL plus ribavirin [8]. Indeed, in that cohort 112 patients with TE results >20 KPa and 97 patients with PLT counts <100,000/mm^3^ were treated without ribavirin. SVR12 rates of 91.0% and 94.7% were registered in these subgroups, respectively. These rates are in keeping with the 99.1% and 96.2% registered in the corresponding subgroups from our cohort. Moreover, in our study SVR12 rates were confirmed to be high in patients with evidence of portal hypertension, either endoscopic (98.2%) or ultrasonographic (97.1%).

The majority of our patients had cirrhosis of CTP class A, therefore they cannot be compared to those with decompensated cirrhosis included in the ASTRAL-4 study focusing on CTP B patients and supporting the addition of ribavirin for 12 weeks of treatment [12]. However, patients with TE >20 KPa are expected to have a less favorable prognosis than patients with lower results, due to the high risk of portal hypertension associated with this TE result.

Our data give reason to implement the strategy to treat all compensated cirrhotic patients without ribavirin, including those with F1–F2 varices or hypertensive gastropathy, and avoid unnecessary pre-treatment genotyping based on the high SVR12 achieved in this GT3 cohort of patients with advanced cirrhosis. Very recent results have shown that, while the decompensated cirrhosis outcome was not significantly ameliorated by DAA treatment, patients with compensated cirrhosis who achieved sustained virologic response after DAA treatment experienced similarly decreased mortality and cancer risk decline as compared to patients with chronic hepatitis [20]. Of note, evidence that the absence of portal hypertension can be attained by patients achieving favorable Baveno VI status after SVR12 was supported by the analysis of data from a large cohort of patients with HCV- or HBV-associated cirrhosis used to validate Baveno VI criteria (Consensum Workshop Criteria) [21].

In the era of DAA, patients with GT3 and cirrhosis are also considered difficult to treat with Glecaprevir and Pibrentasvir (G/P). In the SURVEYOR-2 study, 12 weeks of treatment led to an SVR12 rate of 98% in treatment naïve patients, while 16 weeks were required to achieve an SVR12 of 96% in treatment experienced patients [22]. In a real-life experience, including GT3 cirrhotic patients from the Veterans cohort, evidence of an SVR12 of 94% was provided by Belperio et al. with Glecaprevir/Pibrentasvir (G/P) for 12 weeks. [23]. Moreover, in patients with advanced cirrhosis and high mortality risk, a regimen not including a protease inhibitor should be preferred.

Despite its small sample size, this study included very well-characterized patients either in terms of liver disease or in terms of co-morbidities from alcohol abuse. Given the SVR12 of 99.1%, the lack of a control group of patients receiving ribavirin does not appear to be a limiting factor. On the other hand, the number of patients with cirrhosis is currently declining after 6 years of availability of oral DAA; moreover, test and treat strategies using an STR as SOF/VEL can now be easily and safely adopted regardless of genotype, fibrosis and even portal hypertension.

A limitation of our study might be the lack of data on NS5A-associated RASs at baseline. However, according to previous evidence generated by our group showing that baseline NS5A associated RASs are quite rare in our geographical area [7], no further baseline RASs testing is routinely performed in naïve patients and in patients who failed Peg-Interferon and ribavirin before SOF/VEL treatment. This evidence is also supported by a number of studies performed in other European countries [5,24].

Simplified treatment is currently recommended by the WHO in order to reach HCV elimination by 2030 [25]. GT3 patients are particularly frequent among intravenous drug users considered at high risk of HCV infection transmission and are an important reservoir for the infection [26]. We suggest that even GT3 patients with cirrhosis, prior Peg-Interferon and ribavirin treatment failure history and evidence of portal hypertension can safely start SOF/VEL treatment with any testing for resistance.

## 5. Conclusions

In conclusion, this study shows the effectiveness of STR based on SOF/VEL without the addition of ribavirin in cirrhotic patients with 10% 5-year risk of mortality, as patients with evidence of portal hypertension. The high SV12 rate and the absence of any treatment-related SAEs suggest that this regimen can be adopted without risk in the perspective of a test and treat strategy required to achieve WHO 2030 goals.

## Figures and Tables

**Table 1:** Baseline characteristics of patients overall and by transient elastography (TE) results ≥ or <20 KPa.

	Overall 205	≥20 KPa 111	<20 KPa 94
Mean age (years) ±SE range	52.9 ± 8.7 (25–85)	53.4 ± 6.9 (35–78)	52.2 ± 9.9 (27–85)
Male, n (%)	175 (85.4)	100 (90.1)	75 (79.8)
Genotype 3a vs. 3b	204 (99.0)	110 (99.0)	93 (99.0)
Mean HCV RNA log10 ± SE (IU/mL)	2.53 ± 4.30	2.58 ± 5.01	2.66 ± 3.54
Mean ALT U/L (range)	93.27 (14–366)	91.17 (15–252)	96.42 (25–366)
Mean TE results in KPa (range)	22.8 (12.5–75)	28.6 (20–75)	14.9 (12.5–19.1)
Mean platelet count in cells/mm^3^	137,000 (16–384)	115,000 (16–384)	161,000 (63–301)
Pts with PLT count <150,000 mm^3^	133 (64.9)	90 (81.0)	43 (45.3)
Mean albumin level ±SE (g/dl)	3.99 ± 0.51	3.89 ± 0.52	4.13 ± 0.47
Pts with albumin level <3.5 g/dl	30 (14.6)	27 (24.3)	3 (3.20)
Mean APRI ±SE	2.12 ± 1.58	2.4 ± 1.62	1.86 ± 1.57
Mean FiB-4 ±SE	4.4 ± 3.0	5.5 ± 3.5	3.18 ± 1.58
Naïve N (%)	150 (72.8)	75 (67.1)	75 (17.7)
Mean MELD score	8.1 (6–22)	8.7 (6–22)	7.4 (6–14)
CPT class A5 N (%)	162 (79.0)	80 (72.1)	82 (87.2)
Patients with history of past decompensation	8 (3.9)	8 (7.2)	0
Varices or hypertensive gastropathy N (%)	63 (30.7)	62 (55.9)	1 (1.1)
Patients with spleen diameter >15 cm or PV diameter >15 mm	35 (17.1)	29 (26.1)	7 (7.4)
Ascites N (%)	8 (3.9)	n.a.	n.a.
HCC N (%)	10 (5.3)	10 (9.0)	0
Combined portal hypertension evidence by presence of US signs, platelets <150,000 µL	147 (71.7)	103 (92.7)	44 (46.8)
Diabetes N (%)	36 (17.6)	22 (19.8)	14 (14.9)
Alcohol abuse N (%)	38 (18.5)	25 (22.5)	13 (13.8)
HIV positive N (%)	20 (9.8)	11 (9.9)	9 (9.6)
Past IDU	30 (14.6)	16 (14.4)	14 (14.9)

HCV: Hepatitis C Virus; Pts: platelets; APRI: AST to platelet ratio index; FIB-4: Fibrosis-4; MELD: Model For End-Stage Liver Disease; CPT: Child Pugh Turcotte; HCC: Hepatocellular Carcinoma; US: ultrasound; IDU: intravenous drug use.

**Table 2:** Characteristics of patients with relapse (Viremia levels were defined as high if they were higher than 1,000,000 IU/mL.

Pt No.	Gender	Age	High Viral Load	Past IDU	HIV-Positive	HCV Subtype	CTP Class	Portal Hypertension	Tx History
1	M	32	yes	no	no	a	A	no	prior treatment
2	M	37	yes	no	no	a	A	no	naïve
3	M	71	yes	no	no	a	A	yes	naïve
5	F	71	yes	yes	no	a	A	no	prior treatment
4	M	57	no	no	yes	a	A	no	prior treatment

Pt: patient; CTP: Child-Turcotte-Pugh class of cirrhosis; IDU: intravenous drug users; Tx: treatment.
